# Noninvasive evaluation of ^18^F-FDG/^18^F-FMISO-based Micro PET in monitoring hepatic metastasis of colorectal cancer

**DOI:** 10.1038/s41598-018-36238-x

**Published:** 2018-12-13

**Authors:** Mingyu Zhang, Huijie Jiang, Rongjun Zhang, Hailong Xu, Hao Jiang, Wenbin Pan, Xin Li, Yiqiao Wang, Song Wang

**Affiliations:** 1 Department of Radiology, The Second Affiliated Hospital of Harbin Medical University, Harbin, China; 2 Jiangsu Institute of Nuclear Medicine, Wuxi, China; 3 Department of Radiology, Longhua Hospital, Shanghai University of Traditional Chinese Medicine, No. 725, South Wanping Road, Shanghai, 200032 China

## Abstract

This study aimed to explore the application of two radiotracers (^18^F-fluorodeoxyglucose (FDG) and ^18^F-fluoromisonidazole (FMISO)) in monitoring hepatic metastases of human colorectal cancer (CRC). Mouse models of CRC hepatic metastases were established by implantation of the human CRC cell lines LoVo and HT29 by intrasplenic injection. Wound healing and Transwell assays were performed to examine cell migration and invasion abilities. Radiotracer-based cellular uptake *in vitro* and micro-positron emission tomography imaging of liver metastases *in vivo* were performed. The incidence of liver metastases in LoVo-xenografted mice was significantly higher than that in HT29-xenografted ones. The SUVmax/mean values of ^18^F-FMISO, but not ^18^F-FDG, in LoVo xenografts were significantly greater than in HT29 xenografts. *In vitro*, LoVo cells exhibited stronger metastatic potential and higher radiotracer uptake than HT29 cells. Mechanistically, the expression of HIF-1α and GLUT-1 in LoVo cells and LoVo tumor tissues was remarkably higher than in HT29 cells and tissues. Linear regression analysis demonstrated correlations between cellular ^18^F-FDG/^18^F-FMISO uptake and HIF-1α/GLUT-1 expression *in vitro*, as well as between ^18^F-FMISO SUVmax and GLUT-1 expression *in vivo*. ^18^F-FMISO uptake may serve as a potential biomarker for the detection of liver metastases in CRC, whereas its clinical use warrants validation.

## Introduction

Colorectal cancer (CRC) is one of the most commonly diagnosed cancers worldwide, accounting for 10% of all malignancies^1,2^. Despite advancement in CRC treatment, liver metastasis remains a great obstacle in effective CRC treatment^3,4^. Up to 25% of patients with primary CRC present with synchronous colorectal liver metastases (CLM) at initial diagnosis, and 50% of patients develop metachronous CLM within 3 years following resection of the primary lesion^5,6^. Liver metastasis is a more important risk factor for survival than the primary CRC itself^7^. Therefore, early detection of CLM is of critical medical interest.

Positron emission tomography (PET) is a non-invasive imaging method that facilitates imaging of the whole tumor and is a promising method to analyze intratumoral heterogeneity^8,9^. Of note, ^18^F-fluorodeoxyglucose (^18^F-FDG) and ^18^F-fluoromisonidazole (^18^F-FMISO) PET is becoming increasingly popular to monitor the biological behavior of tumors, including glucose metabolism, cell proliferation, and hypoxic response^10–12^. ^18^F-FDG is a glucose analogue that can be transported into cells by glucose transporters, where it is phosphorylated to ^18^F-FDG-6-phosphate which cannot undergo further metabolism and remains trapped in the cells. Increased glycolysis or energy metabolism in tumor cells results in an enhancement of ^18^F-FDG uptake and accumulation, an index of tumor growth and development^13,14^. Whereas, ^18^F-FMISO could selectively bind to hypoxic cells, serving as an assessment of hypoxic response in human cancers. ^18^F-FDG/^18^F-FMISO-based PET imaging is used in tumor staging and follow-up examination of multiple malignancies including glioblastoma^15^, head and neck cancer^16^, lung cancer^17^, breast cancer^18^, and CRC^19^. Nevertheless, these radiotracers have not been applied to monitor CLM yet.

This study aimed to investigate whether ^18^F-FDG- and ^18^F-FMISO-based PET can be used to monitor the CLM derived from the CRC cell lines LoVo and HT29, which have different metastatic potentials, by assessing the correlation between radiotracer uptake and tumor marker expression *in vitro* and *in vivo*.

## Methods

### Synthesis of ^18^F-FDG and ^18^F-FMISO

^18^F-FDG and ^18^F-FMISO were synthesized using a multi-functional module (purity >95%, WuXi Jiangyuan Industrial Technology and Trade Corporation) as previously described^20^. The ^18^F-labeled tracers were formulated in normal saline and passed through a 0.22 μm Millipore filter (Millipore, Billerica, MA, USA) for administration to animals.

### Cell culture

The human CRC cell lines LoVo and HT29 were purchased from the Type Culture Collection of the Chinese Academy of Sciences, Shanghai, China, and were maintained in Dulbecco’s Modified Eagle Medium (DMEM; Gibco Corporation, USA) and McCoy’s 5 A medium (Gibco), respectively. The culture media were supplemented with 10% fetal bovine serum (FBS; Hyclone, USA) and 1% penicillin-streptomycin in a humidified atmosphere of 5% CO_2_ at 37 °C. To induce hypoxic response and starvation, the cells were cultured in a humidified atmosphere of 1% O_2_, 5% CO_2_ and 94% N_2_ at 37 °C for 24 h and free-serum starved at 37 °C for 12 h, respectively.

### CLM model

Forty 5-week-old female BALA/C nude mice weighing 16–18 g were purchased from the Animal Laboratory of Cavens Corporate of Changzhou (Changzhou, Jiangsu, China). The mice were maintained and used according to the institutional guidelines of Jiangsu Institute of Nuclear Medicine Animal Care and Use Committee (IACUC). All animal experiments were conducted in compliance with the protocols approved by the Institutional Animal Care and Use Committee (IACUC) of Jiangsu Institute of Nuclear Medicine. All mice were acclimatized for three days and then injected under general anesthesia (2% isoflurane in pure O_2_) with 5.0 × 10^6^ LoVo or HT29 cells (20 mice for each cell line) in 0.15 mL of phosphate-buffered saline (PBS) into the spleens. After injection, body weight was recorded every 3 days.

### Wound healing assay

LoVo or HT29 cells were seeded in 6-well plates at 5 × 10^5^ cells/ml and were cultured until 90% confluency. The cells were scratched with a 10 μL pipette tip to create a wound, followed by imaging (0 h). The cells were then treated with 2% FBS-containing medium and imaged (Olympus, Japan) 24 and 48 h after scratching. The wounded area was measured using the Image J software (NIH, Bethesda, MD, USA). Wound healing was determined using the following formula: wound healing% = (wounded area at 0 h-wounded area at indicated time)/wounded area at 0 h × 100%.

### Cell invasion assay

The cell invasion assay was performed in 24-well Transwell chambers (Corning, Costar, USA)^21^. The upper chambers of the Transwell plates were coated with 20 μL of Matrigel matrix (Corning, 356234, USA) diluted in serum-free DMEM (1:40) and incubated at 37 °C for 1 h. The cells (100 μL, 2 × 10^4^ cells/mL) in serum-free DMEM were added to the upper chamber. Complete medium (500 μL) containing 10% FBS was added to the lower chamber as chemo-attractant. After incubation for 72 h, non-invading cells on the surface of the upper chamber were removed with a cotton swab. The invading cells on the lower membrane were fixed with formaldehyde and stained with Giemsa (G1010, Solarbio, China). The images were taken under an Olympus BX53 microscope at 40× magnification (Olympus, Japan). The cells were counted using the Image J software (NIH).

### *In vitro* cellular uptake of radiotracers

The LoVo and HT29 cells were cultured at 37 °C in a hypoxic atmosphere of 1% O_2_ for 24 h prior to ^18^F-FMISO uptake or in corresponding serum-free medium at 37 °C atmosphere of 5% CO_2_ for 12 h prior to ^18^F-FDG uptake. The cell density was adjusted to 5 × 10^5^ cells/100 µl. For the X group, 100 µl of cell suspension were incubated with the same volume of radiotracer (^18^F-FDG or ^18^F-FMISO) and buffer (DMEM containing 0.2% bovine serum albumin) in a glass tube at 37 °C for 30, 60, 120 and 240 min. The mixture of 100 μl of radiotracer and 200 μl of buffer was used as a control group (the O group). A volume of 100 μl of radiotracer was used to measure the radiotracer doses (the T group). After incubation, the O and X groups were centrifuged at 1000 rpm/min for 5 min and the supernatants were removed. The radioactivity was measured using a PerkinElmer 2480 automatic gamma counter (Waltham, MA, USA). The ratio of cellular radionuclide uptake was calculated using the following formula: X(cpm)-O(cpm)/T(cpm)%. cpm: counts per minute. The experiments were independently repeated three times.

### Micro-PET imaging

^18^F-FDG/^18^F-FMISO-based PET imaging was performed 7 weeks after the injection of tumor cells. The mice were fasted but allowed access to drinking water for 12 h, followed by administration of ^18^F-FDG. ^18^F-FDG and ^18^F-FMISO injections were performed on separate days, 48 h apart. Ten-minute static ^18^F-FDG (3.7 MBq, 100 μCi) and ^18^F-FMISO (14.8 MBq, 400 μCi) PET images were acquired at 1 and 4 h after injection via the tail vein under isoflurane anesthesia, in a 3-dimensional mode using an Inveon micro-PET scanner (Siemens Medical Solutions, Erlangen, Germany). Body temperature of the mice was maintained using a heat lamp. PET images were reconstructed using the Inveon Acquisition Workplace software (version 2.0, Siemens Preclinical Solutions) and an ordered-subset expectation maximization method with the following parameters: matrix, 128 × 128 × 159; voxel size, 0.86 × 0.86 × 0.8 mm; β-value, 1.5, with uniform resolution. The regions of interest (ROI) were drawn on images around the entire liver metastastic lesions using the ASI Pro VM 6.8.6.9 software (Concorde Microsystems, Knoxville, TN, USA). For the semi-quantitative evaluation of ^18^F-FDG or ^18^F-FMISO uptake in the liver metastasis lesions, the highest and the average tracer concentrations were determined as a maximum/mean standardized uptake value (SUVmax/mean) calculated as: SUV_max/mean_ = [Max/Mean × 8000μCi/ml × weight(g)]/Injected dose μCi. The liver metastasis were confirmed by visualization post anatomy.

### Immunofluorescence assay

LoVo and HT29 cells were plated on sterile slides in 6-well plates and cultured in a modular incubator chamber at 37 °C in a hypoxic atmosphere of 1% O_2_ for 24 h prior to staining of HIF-1α and were serum-starved for 12 h prior to staining of glucose transporter 1 (GLUT-1). Cells were washed with PBS three times and fixed with 4% paraformaldehyde for 30 min at room temperature, followed by incubation with 1% Triton X-100 for 15 min at room temperature. Following 30 min of blocking with 10% normal goat serum, the cells were incubated with anti-HIF-1α (1:100; H1alpha67; Abcam, UK) or anti-GLUT-1 (1:100; ab40084; Abcam, UK)^22^. Images were taken with a confocal microscope (C2si; Nikon, Japan). The protein expression was quantified using the Live Cell Imaging System (NIS-Elements, Japan).

### Immunohistochemical staining

The liver metastasis specimens of the xenografts were fixed in 10% formalin for 48 h, paraffin-embedded, and cut into 3-μm-thick sections. Immunohistochemical staining was performed as previously described^23^. Briefly, the slides were incubated with anti-HIF-1α (1:100; Abcam) or anti-GLUT-1 (1:100; Abcam) overnight at 4 °C. The slides were incubated with HRP-labeled goat anti-mouse secondary antibody (Boster, Wuhan, China) for 1 h at room temperature followed by counterstaining with hematoxylin. The staining was observed under a BX53 Olympus microscope (Olympus) at magnification 200×. The brown-yellow staining levels of the HIF-1α and GLUT-1 proteins were evaluated using the Image J software (NIH, Bethesda, MD, USA) and expressed as mean optical density.

### Western blot

Western blot experiments were performed as previously described^24^. Tumor tissues were lysed in RIPA buffer for 30 min at 4 °C. Protein concentrations were determined using a bicinchoninic acid kit (Beyotime Biotech, China). Equivalent amounts of protein were resolved in 10% or 12% SDS-PAGE gel and transferred to polyvinylidene fluoride membranes (Millipore). The membranes were blocked with 10% non-fat dry milk in TRIS-buffered saline containing 0.1% tween-20 for 1 h, and incubated with anti-HIF-1α (1:1000; Abcam), anti-GLUT-1 (1:1000; Abcam), or anti-GAPDH (1:5000; ZhongShan Golden Bridge Biotech Co., China) overnight at 4 °C, followed by additional incubation with peroxidase-conjugated secondary antibody (1:5000; ZhongShan Golden Bridge Biotech Co., China) for 1 h at room temperature. The bands were detected using a Bio-Rad chemiDoc XRS + imaging system (Bio-Rad, Hercules, CA, USA) and quantified using Image J (NIH) by normalization to GAPDH.

### Statistical analysis

All data were expressed as means ± standard deviation. Statistical analysis was performed using SPSS version 19 (IBM, Armonk, NY, USA). The differences between the two groups were assessed using the Student’s unpaired *t*-test. The differences in liver metastatic potential were compared by the Fisher exact test. The correlations were analyzed using linear regression. A *P* value < 0.05 was considered statistically significant.

## Results

### LoVo cells are more prone to metastasis than HT29 cells *in vivo*

**Figure 1:**
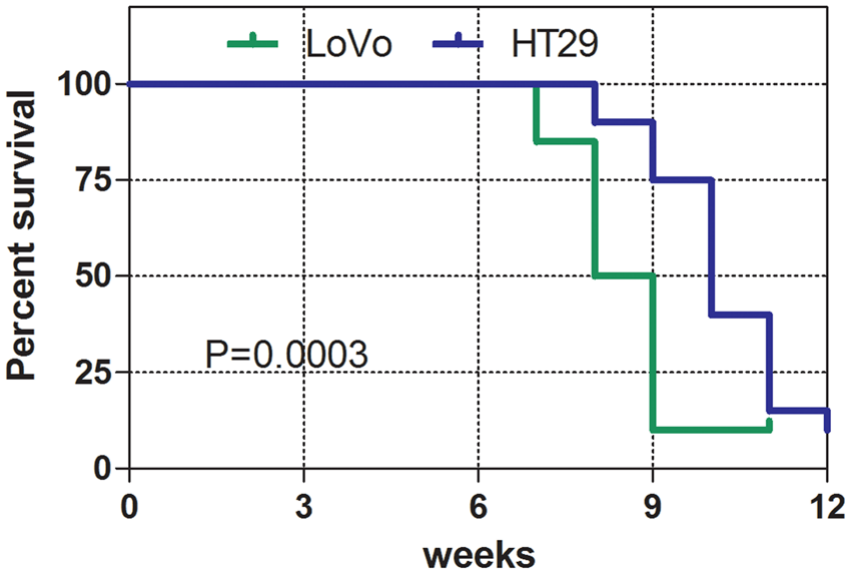
Survival curves of the LoVo and HT29 xenograft models. **P < 0.01; *n* = 20.

To evaluate the metastatic potential of CRC cells *in vivo*, we established two xenograft models using LoVo and HT29 cells, and compared the ratio of liver metastases between these two models. Liver metastases were confirmed by pathological examination. The percentage of liver metastases was defined as the number of mice with liver metastasis in a group divided by the total number of mice in the group. The percentage of liver metastases with LoVo cells was significantly higher than with HT29 cells (10/20, 50% vs. 4/20, 20%, *P* = 0.048). The median survival of LoVo-CLM mice was significantly shorter than that of the HT29-xenografted ones (8.5 weeks *vs*. 10.5 weeks, *P* < 0.01; Fig. 1). These data suggest that LoVo cells exhibit a stronger metastatic potential than HT29 cells.

### Comparison of *in vivo* radiotracer uptake between LoVo and HT29 CLM

**Figure 2:**
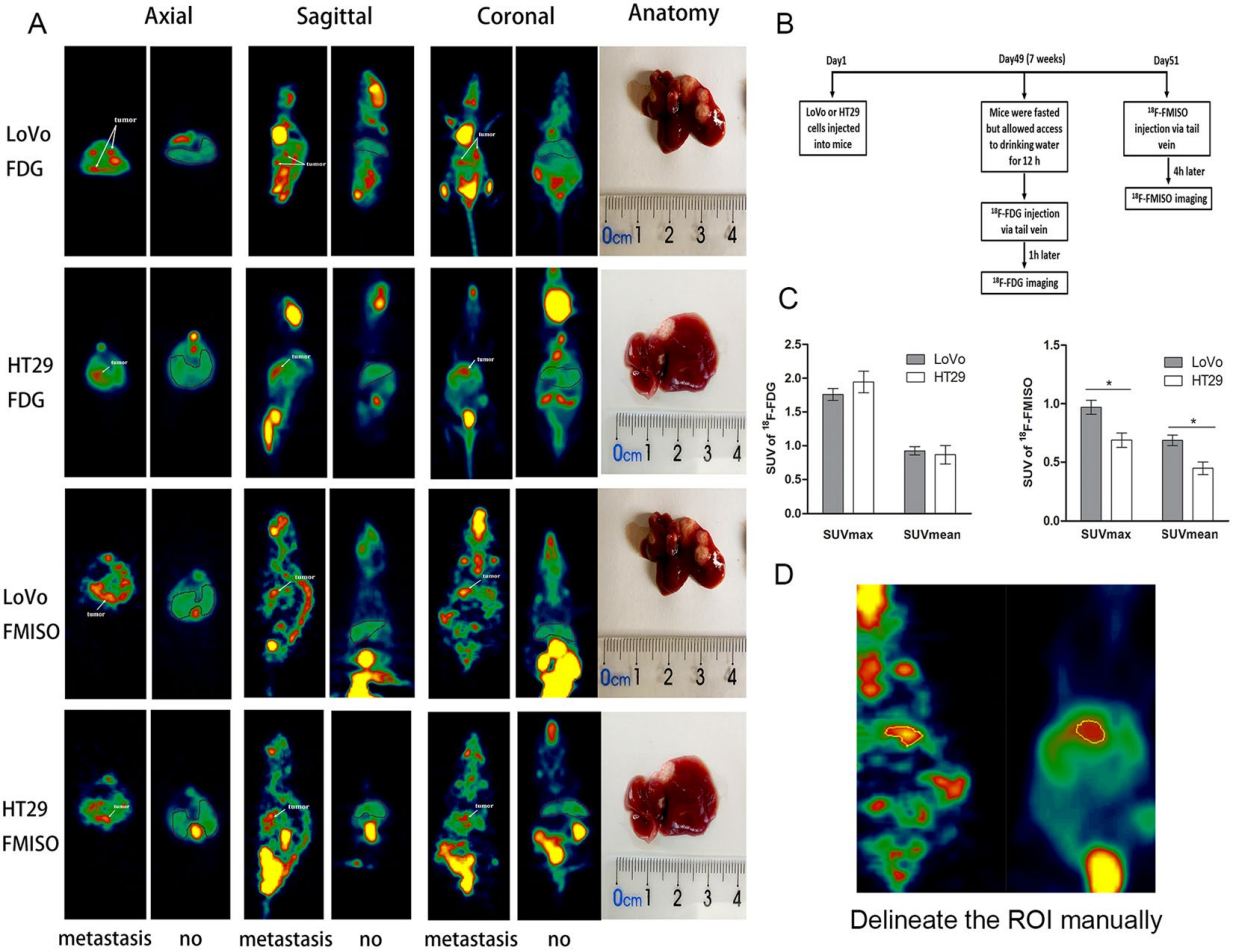
(**A**) ^18^F-FDG/^18^F-FMISO-based micro-positron emission tomography (PET) images of liver metastases of LoVo and HT29 xenografts 7 weeks after injection with corresponding tumor cells. The gray outline area of the PET images of the mice with no liver metastasis represents the liver area. White arrows indicate the uptake of radiotracers in tumor tissues. (**B**) The timeline of micro-PET. (**C**) Quantification of micro-PET regions of interest analysis (ROI) of ^18^F-FDG and ^18^F-FMISO uptake in xenograft tumor tissues. (**D**) Demonstration of ROI delineation. *P < 0.05, n = 10 (LoVo) and n = 4 (HT29).

We next sought to investigate whether radiotracer uptake could serve as an indicator of CRC metastasis. To evaluate and compare the abilities of radiotracer uptake *in vivo* between LoVo and HT29 xenografts, we performed a ^18^F-FDG PET/^18^F-FMISO-based Micro-PET analysis using metastatic liver tissues from LoVo and HT29 CLMs, respectively. The timeline of PET imaging is shown in Fig. 2B. The location of liver and tumor tissues in PET imaging were corresponded with the anatomical location. Liver metastasis >0.5 cm could be detected in ^18^F-FDG PET/^18^F-FMISO-based micro-PET images. The images of ^18^F-FDG PET and ^18^F-FMISO PET as well as the images of liver metastases from LoVo or HT29 xenograft models are shown in Fig. 2A. The SUVmax and SUVmean values of ^18^F-FDG in normal liver tissue (the mice without metastatic lesions) was 0.25 ± 0.02 and 0.22 ± 0.02, and the SUVmax and SUVmean values of ^18^F-FMISO in normal liver tissue was 0.17 ± 0.01 and 0.15 ± 0.007. Based on the SUVmax and SUVmean values of ^18^F-FDG, there was no significant difference between LoVo and HT29 tumor tissues (*P* = 0.683 and 0.339, respectively) (Table 1 and Fig. 2B). Moreover, both SUVmax and SUVmean values of ^18^F-FMISO in LoVo tumors were significantly greater than those of HT29 tumors (0.96 ± 0.189 *vs*. 0.67 ± 0.104 and 0.68 ± 0.139 *vs*. 0.41 ± 0.102, respectively; *P* = 0.001 and <0.00#, respectively) (Fig. 2C), suggesting that ^18^F-FMISO, but not ^18^F-FDG, may serve as an indicator of hepatic metastasis in CRC.

**Table 1:** Comparison of ^18^F-FDG and ^18^F-FMISO SUV indices between LoVo and HT29 xenograft tumor tissues.

	n	SUV (max)	SUV (mean)
^18^F-FDG PET			
Lovo	10	1.75 ± 0.276	0.92 ± 0.196
HT29	4	1.81 ± 0.308	0.82 ± 0.242
P value		0.683	0.339
^18^F-FMISO PET			
LoVo	10	0.96 ± 0.189	0.68 ± 0.139
HT29	4	0.67 ± 0.104	0.41 ± 0.102
P value		0.001	<0.001

### LoVo cells have higher migratory and invasive abilities than HT29 cells *in vitro*

**Figure 3:**
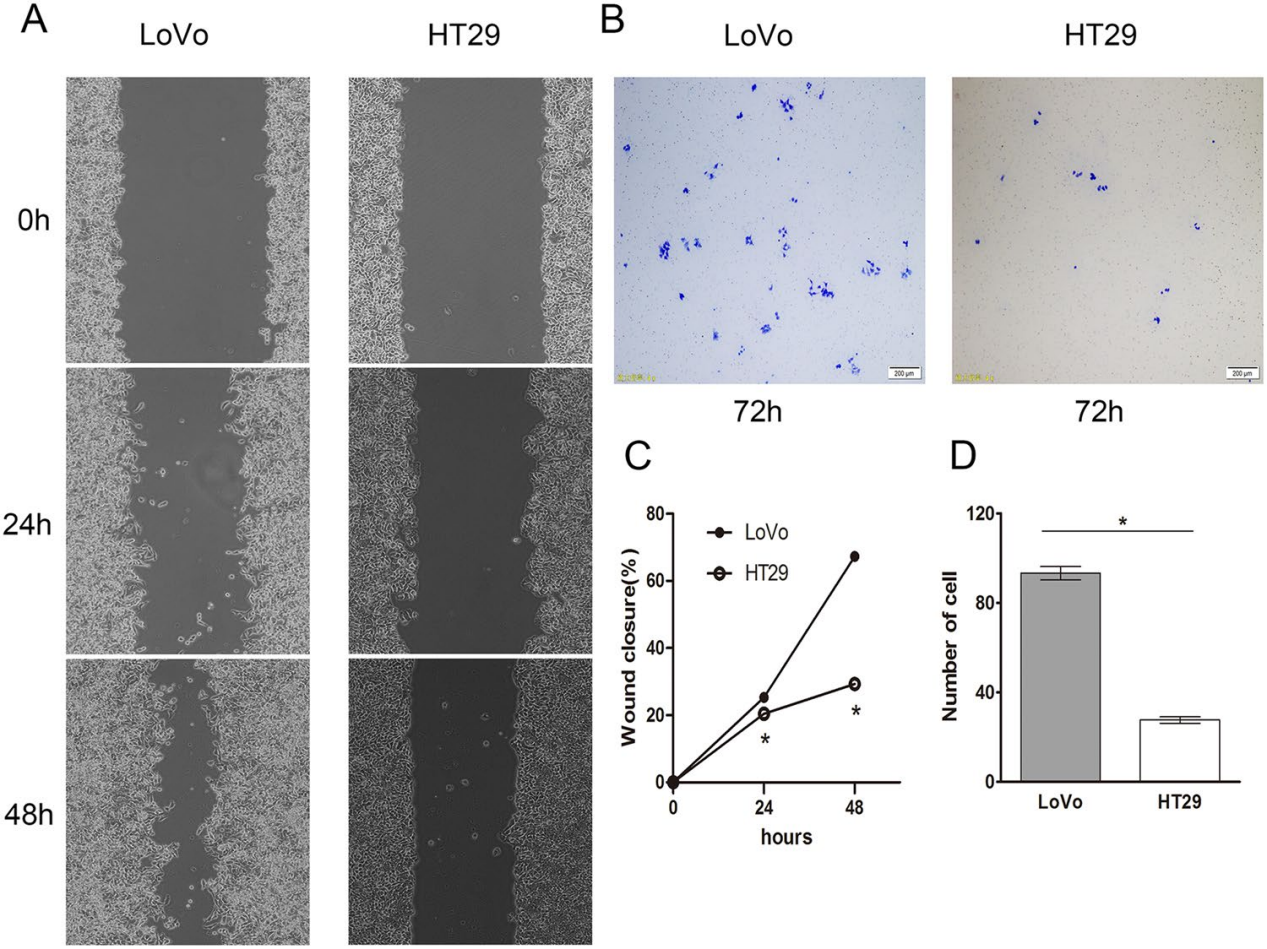
(**A**) LoVo and HT29 cell monolayers were scratched using pipette tips. The images were acquired at 0, 24, and 48 h after wound formation. Magnification 100×. (**B**) LoVo and HT29 cells were subjected to a Matrigel extracellular matrix cell invasion assay. The cells were allowed to invade for 72 h. The invading cells were stained with Giemsa and observed under a microscope at a magnification of 40×. (**C**) Quantification of cell wound closure (%) of LoVo and HT29 cells. (**D**) Quantification of cell invasion assay. *P < 0.05; *n* = 3.

To reveal the molecular mechanism underlying the differences of metastatic potential and the abilities of radiotracer uptake between LoVo and HT29 cells *in vivo*, we performed wound healing and transwell assays to compare the migratory and invasive abilities of LoVo and HT29 cells *in vitro*^25^. As shown in Fig. 3A,C, the percentages of wounded healing area in LoVo cells at different time points were greater than those in HT29 cells (25.27 ± 0.6% vs. 20.46 ± 0.57% at 24 h and 67.3 ± 0.51% vs. 29.32 ± 0.43% at 48 h, respectively; *P* < 0.05). In addition, the number of migrating LoVo cells was significantly higher than that of HT29 cells (93.3 ± 5.13 vs. 27.6 ± 2.51, *P* < 0.05; Fig. 3B,D). These data suggest that LoVo cells are more prone to migration and invasion than HT29 cells *in vitro*.

### *In vitro* cellular radiotracer uptake analysis

**Figure 4:**
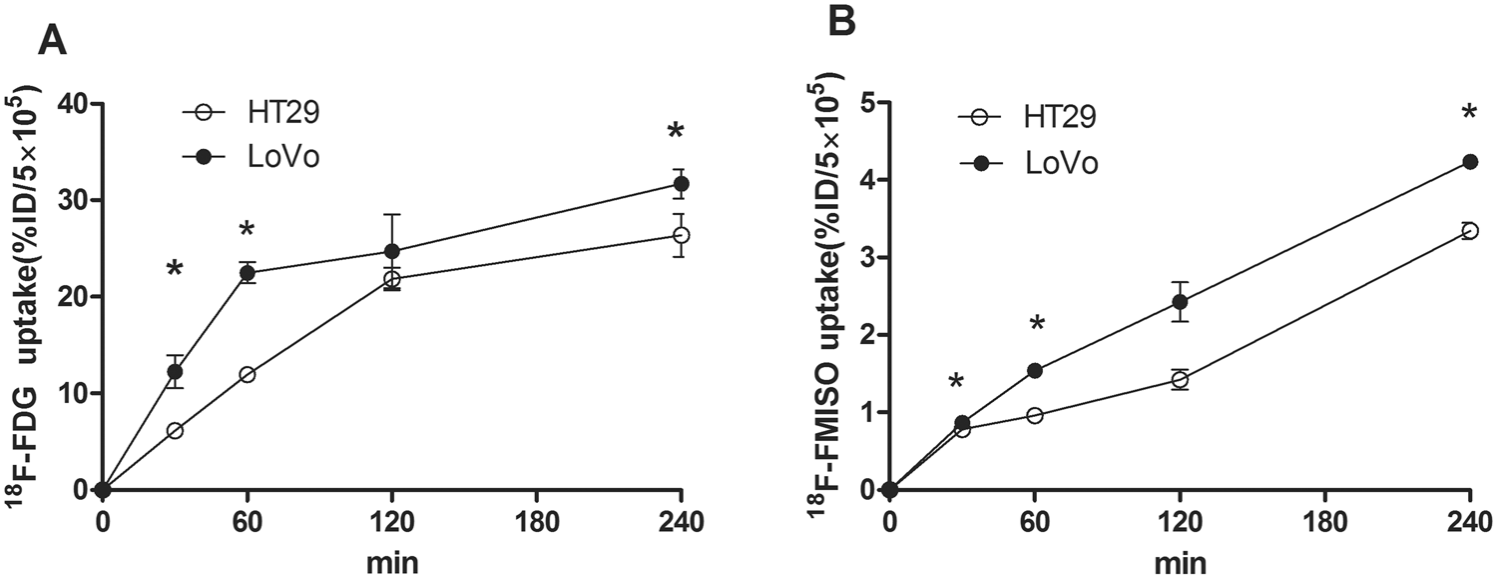
Time courses of *in vitro* cellular uptake of ^18^F-FDG/^18^F-FMISO in LoVo and HT29 cells. Cells were incubated with ^18^F-FDG or ^18^F-FMISO for 30, 60, 120, and 240 min. Data are expressed as means ± standard deviation of the percentage of the injected dose (%ID) of ^18^F-FDG (A) or ^18^F-FMISO (**B**) per 5 × 10^5^ cells. *P < 0.05; *n* = 3 (LoVo VS HT29).

To evaluate whether the abilities of radiotracer uptake in CRC cells were correlated with their metastatic potential, we performed time course analyses of cellular radiotracer uptake in LoVo and HT29 cells. As shown in Tables 2, 3 and Fig. 4, both ^18^F-FDG and ^18^F-FMISO were taken up into LoVo and HT29 cells in a time-dependent manner. At 30, 60, and 240 min, the uptake rates of both ^18^F-FDG (Fig. 4A) and ^18^F-FMISO (Fig. 4B) in LoVo cells were significantly higher than those in HT29 cells (for ^18^F-FDG, *P* = 0.003, 0.003, and 0.026, respectively; for ^18^F-FMISO, *P* = 0.049, <0.001, and 0.002, respectively). These results indicate that LoVo cells are more capable of taking up radiotracers than HT29 cells, suggesting that the ability of radiotracer uptake may be positively correlated with the metastatic potential of CRC cells.

**Table 2:** Comparison of *in vitro* uptake of ^18^F-FDG between LoVo and HT29 cells (%).

	LoVo	HT29	P
30 min	12.24 ± 1.69	6.15 ± 0.075	0.003
60 min	22.49 ± 1.072	11.96 ± 0.2	0.003
120 min	24.71 ± 3.8	21.85 ± 1.178	0.282
240 min	31.7 ± 1.509	26.36 ± 2.218	0.026

RMANOVA (time*groups), *P* = 0.000; t test (LoVo vs. HT29): *P = *0.003, 0.003, 0.026 at 30, 60, 240 min, respectively.

**Table 3:** Comparison of *in vitro* uptake of ^18^F-FMISO between LoVo and HT29 cells (%).

	LoVo	HT29	P
30 min	0.87 ± 0.05	0.78 ± 0.06	0.049
60 min	1.54 ± 0.08	0.96 ± 0.05	0.000
120 min	2.06 ± 0.549	1.31 ± 0.168	0.087
240 min	4.23 ± 0.128	3.34 ± 0.181	0.002

RMANOVA (time*groups), P = 0.001; t test (LoVo vs HT29), P = 0.049, 0.000, 0.002 at 30, 60, 240 min, respectively.

### HIF-1α and GLUT-1 were induced more efficiently in LoVo cells than in HT29 cells

**Figure 5:**
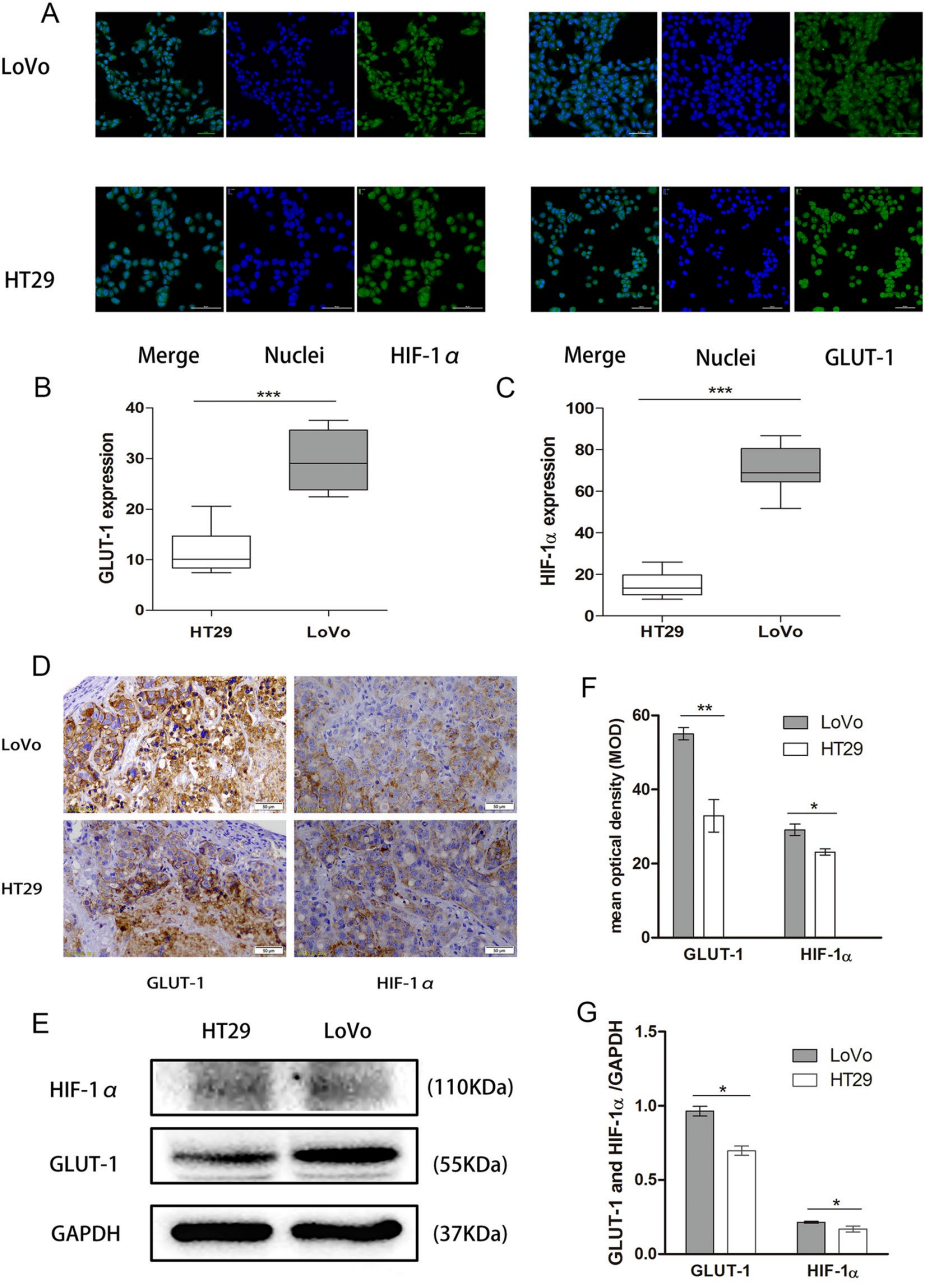
Comparison of induction of HIF-1α and GLUT-1 between LoVo and HT29 cells/tumor tissues. (**A**) Micrographs of immunofluorescence staining for nuclei (blue) and HIF-1α/GLUT-1 (green) in LoVo (upper panel) and HT29 (lower panel) cells (magnification, 200×). (**B**) and (**C**) Quantification of HIF-1α and GLUT-1 staining using Live cell Imaging System. (**D**) Immunohistochemical staining for GLUT-1/HIF-1α (brown) in LoVo (upper panel) and HT29 (lower panel) xenograft tumor tissues (magnification, 200×), respectively. (**E**) Western blot analyses of HIF-1α and GLUT-1 protein expression in LoVo (right lane) and HT29 (left lane) xenograft tumor tissues. (**F**) Quantification of immunohistochemical staining. (**G**) Quantification of Western blot analyses. ***P < 0.0001, **P < 0.001, *P < 0.05, *n* = 3.

Since HIF-1α and GLUT-1 are closely associated with cancer metastasis, we next sought to determine and compare the expression of HIF-1α and GLUT-1 in LoVo and HT29 cells. As shown in Fig. 5, there was a stronger fluorescent staining of hypoxia-induced HIF-1α or serum starvation-induced GLUT-1 in LoVo cells than that in HT29 cells (71.06 ± 11.24 *vs*. 14.98 ± 5.74 and 29.3 ± 5.63 *vs*. 10.95 ± 4.47, *P* < 0.0001). In addition, the positive area percentage of GLUT-1 expression in LoVo and HT29 tumor tissues were 21.6% and 12.9%, respectively. The positive area percentage of HIF-1α expression in LoVo and HT29 tumor tissues were 11.4% and 9.1%, respectively. GLUT-1 and HIF-1α proteins were strongly stained in LoVo tumor tissues compared with HT29 tumor tissues (55.1 ± 2.85 *vs*. 32.94 ± 7.62 and 29.14 ± 2.63 *vs*. 23.13 ± 1.43, respectively, *P* < 0.05) measured by immunohistochemistry and expressed as mean density (Fig. 5D). Consistently, the results showed that the protein levels of both HIF-1α and GLUT-1 in LoVo tumor tissues were significantly higher than those in HT29 tissues (0.21 ± 0.006 *vs*. 0.16 ± 0.2 and 0.96 ± 0.03 *vs*. 0.69 ± 0.03, respectively, *P* < 0.05) (Fig. 5E–G). These results indicate that the expression of HIF-1α and GLUT-1 proteins in highly metastatic LoVo cells was induced more efficiently than that in HT29 cells both *in vitro* and *in vivo*.

### Correlation analysis

**Figure 6:**
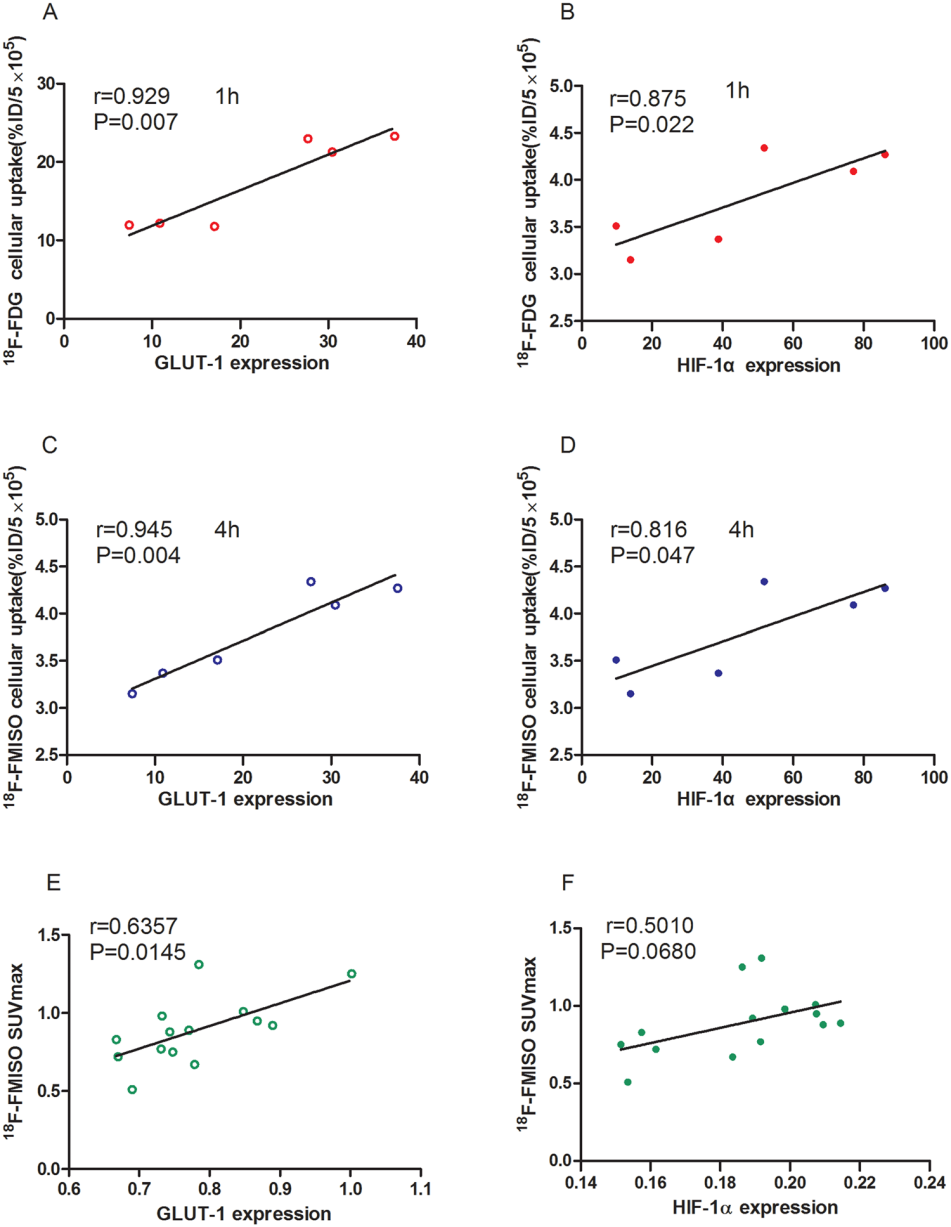
Correlation analyses between various parameters. (**A**) and (**B**) correlation of 18F-FDG uptake with GLUT-1 (**A**) and HIF-1α (**B**) expression *in vitro*. (**C**,**D**) correlation of ^18^F-FMISO uptake with GLUT-1 (**C**) and HIF-1α (**D**) expression *in vitro*. (**E**) and (**F**) correlation of *in vivo* values of ^18^F-FMISO SUVmax with GLUT-1 (**E**) and HIF-1α (**F**) expression in tumor tissues.

We next sought to determine the correlations between radiotracer uptake and HIF-1α/GLUT-1 protein expression. We measured the parameters of radiotracer uptake 1 h post ^18^F-FDG injection and 4 h post ^18^F-FMISO injection, which are the ideal time for radionuclide imaging. The results showed that *in vitro* cellular ^18^F-FDG uptake was significantly correlated with both GLUT-1 and HIF-1α expression (r = 0.929, *P* = 0.007 and r = 0.875, *P* = 0.022, respectively) (Fig. 6A,B), as well as *in vitro* (r = 0.945, *P* = 0.004 and r = 0.816, *P* = 0.045, respectively, Fig. 6C,D). For *in vivo* parameters, only ^18^F-FMISO SUV value had statistically significant differences in LoVo and HT29 liver metastasis tissue; therefore, we performed correlation analysis between ^18^F-FMISO SUVmax value and tumor markers. The result showed that the SUVmax value of ^18^F-FMISO was significantly correlated with GLUT-1 expression (r = 0.6357, *P* = 0.0145, Fig. 6E), but not with HIF-1α expression (r = 0.501, *P* = 0.068) (Fig. 6F).

## Discussion

In the present study, we investigated the use of ^18^F-FDG/^18^F-FMISO-based PET imaging in CLM mouse models and demonstrated that LoVo cells have a stronger metastatic potential than HT29 cells. The underlying mechanisms were also examined. These data suggest ^18^F-FMISO-based PET imaging as a promising method in CLM monitoring.

^18^F-FMISO imaging has been used as a molecular probe in the assessment of hypoxic response in many types of xenograft tumor models including CRC^26^. Previous studies have shown that hypoxia duration is associated with increased risk of invasion and metastasis because of HIF-1α expression and a series of responses caused by HIF-1α expression under hypoxic conditions^27,28^. Therefore, hypoxic imaging can indicate the metastatic potential of cells.

Although Wang *et al*.^29^ have revealed the metastatic characteristics of SW620 and SW480 CRC cell lines using ^18^F-FDG and ^18^F-FLT, it remains unknown whether ^18^F-FDG and ^18^F-FMISO PET imaging may monitor cell glucose metabolism and hypoxic response and may be useful to predict liver metastatic potential. In this study, we used ^18^F-FDG and ^18^F-FMISO PET imaging to monitor the biological behavior and predict the CLM derived from LoVo and HT29 cells. The results demonstrated that the biological behavior of LoVo-derived tumors was significantly different from that of HT29-derived tumors, as evidenced by higher migration and invasion abilities in LoVo cells than in HT29 cells, as well as by shorter median survival of LoVo-CLM mice than that of HT29-CLM mice. These data are consistent with the results of ^18^F-FDG/^18^F-FMISO PET imaging showing that LoVo cells have stronger metastatic potential than HT29 cells. A study from Deng *et al*. also supports our findings^30^.

Tumor-specific markers are important in monitoring occurrence and development of tumors. Under hypoxic conditions, tumors exhibit enhanced glycolysis, angiogenesis invasion, and metastasis by inducing HIF-1α, which in turn promotes relevant gene expression^31^. Thus, detection of HIF-1α and GLUT-1 activity may accurately reflect metabolic ability of tumors or tumor cells. In this study, we examined HIF-1α and GLUT-1 expression and analyzed their correlation with radiotracer uptake. We observed a statistically significant correlation between ^18^F-FDG uptake and GLUT-1/HIF-1α expression, as well as between ^18^F-FMISO uptake *in vitro*. These data also support the correlation between GLUT-1/HIF-1α expression level and tumor metastatic potential.

Nevertheless, unlike the *in vitro* data, our study showed a weak correlation between ^18^F-FMISO SUVmax and GLUT-1 expression, but not HIF-1α. One possible reason is that the liver metastasis nodules were small in mice and the tumor hypoxic regions were also small. In the quantitative analysis of tumor hypoxia, the sensitivity of SUV measured by ROI is not as sensitive as that of bulk solid tumor. Another possible reason is the small number of CLM models. Kudo *et al*. showed that HIF-1α expression is heterogeneous in tumors^32^. In future experimental plans, we will further establish a CRC subcutaneous tumor mice model and increase the number of samples to further explore the correlation between FMISO SUV values and HIF-1 expression in CRC. In a previous study, Sato *et al*.^33^ claimed that the ^18^F-FMISO SUVmax in the primary site of oral squamous cell carcinoma was associated with HIF-1α expression and confirmed that ^18^F-FMISO uptake indicates the presence of hypoxic areas in tumor tissues. On the other hand, a study by Kawai *et al*.^34^ demonstrated that ^18^F-FMISO uptake does not correlate with HIF-1α expression in either newly diagnosed or recurrent malignant glioma patients. The relationship of ^18^F-FMISO uptake and HIF-1α (or GLUT-1) expression needs to be further confirmed. In the future, we plan to compare the differences in PET parameters between primary and metastatic lesions in a variety of animal models and CRC patients, and to study comprehensively the use of PET imaging in the detection and prediction of liver metastases from CRC.

Taken together, the uptake of ^18^F-FDG and ^18^F-FMISO can reflect the different biological behavior in LoVo and HT29 cells *in vitro* and *in vivo*. ^18^F-FMISO uptake may serve as a potential biomarker for detection of liver metastases in CRC, whereas its clinical utilization is warranted to validate

## Electronic supplementary material

Supplementary Information

## References

[1] Torre, L. A. *et al*. Global cancer statistics, 2012. *CA Cancer J Clin* 65:87–108 (2015). 10.3322/caac.21262 25651787

[2] Zheng ZX (2014). Colorectal cancer incidence and mortality in China, 2010. Asian Pac J Cancer Prev.

[3] Edwards BK (2010). Annual report to the nation on the status of cancer, 1975–2006, featuring colorectal cancer trends and impact of interventions (risk factors, screening, and treatment) to reduce future rates. Cancer.

[4] Bosetti C (2011). Recent trends in colorectal cancer mortality in Europe. Int J Cancer.

[5] McMillan, D. C., McArdle, C. S. Epidemiology of colorectal liver metastases. Surgical Oncology-Oxford **16** (2007). 10.1016/j.suronc.2007.04.008 17493802

[6] Manfredi S (2006). Epidemiology and management of liver metastases from colorectal cancer. Ann Surg.

[7] Lam VW (2014). A systematic review of a liver-first approach in patients with colorectal cancer and synchronous colorectal liver metastases. HPB (Oxford).

[8] O’Connor JPB (2017). Cancer heterogeneity and imaging. Semin Cell Dev Biol.

[9] Gatenby RA, Grove O, Gillies RJ (2013). Quantitative imaging in cancer evolution and ecology. Radiology.

[10] Shady W (2016). Metabolic tumor volume and total lesion glycolysis on FDG-PET/CT can predict overall survival after (90)Y radioembolization of colorectal liver metastases: A comparison with SUVmax, SUVpeak, and RECIST 1.0. Eur J Radiol.

[11] Whisenant JG (2013). Reproducibility of static and dynamic (18)F-FDG, (18)F-FLT, and (18)F-FMISO MicroPET studies in a murine model of HER2+ breast cancer. Mol Imaging Biol.

[12] Everitt SJ (2014). Differential (18)F-FDG and (18)F-FLT Uptake on Serial PET/CT Imaging Before and During Definitive Chemoradiation for Non-Small Cell Lung Cancer. J Nucl Med.

[13] Ruers TJ (2002). Value of positron emission tomography with [F-18]fluorodeoxyglucose in patients with colorectal liver metastases: a prospective study. J Clin Oncol.

[14] Fukuda H, Kubota K, Matsuzawa T (2013). Pioneering and fundamental achievements on the development of positron emission tomography (PET) in oncology. Tohoku J Exp Med.

[15] Spence AM (2008). Regional hypoxia in glioblastoma multiforme quantified with [18F]fluoromisonidazole positron emission tomography before radiotherapy: correlation with time to progression and survival. Clin Cancer Res.

[16] Norikane, T. *et al*. Correlation of (18)F-fluoromisonidazole PET findings with HIF-1alpha and p53 expressions in head and neck cancer: comparison with (18)F-FDG PET. *Nucl Med Commun* **35**, 30–5, (2014). 10.1097/MNM.0000000000000010 24121312

[17] Arvold ND (2016). Tumor Hypoxia Response After Targeted Therapy in EGFR-Mutant Non-Small Cell Lung Cancer: Proof of Concept for FMISO-PET. Technol Cancer Res Treat.

[18] Cheng J (2013). 18F-fluoromisonidazole PET/CT: a potential tool for predicting primary endocrine therapy resistance in breast cancer. J Nucl Med.

[19] Marcus C (2015). (1)(8)F-FDG PET/CT and Colorectal Cancer: Value of Fourth and Subsequent Posttherapy Follow-up Scans for Patient Management. J Nucl Med.

[20] Bourdier T (2011). Fully automated one-pot radiosynthesis of O-(2-[18F]fluoroethyl)-L-tyrosine on the TracerLab FX(FN) module. Nucl Med Biol.

[21] Wang Q (2016). Kukoamine A inhibits human glioblastoma cell growth and migration through apoptosis induction and epithelial-mesenchymal transition attenuation. Sci Rep.

[22] Mojic M (2014). Extracellular iron diminishes anticancer effects of vitamin C: an *in vitro* study. Sci Rep.

[23] Ding Z (2010). Expression and significance of hypoxia-inducible factor-1 alpha and MDR1/P-glycoprotein in human colon carcinoma tissue and cells. J Cancer Res Clin Oncol.

[24] Sato M (2015). LW6, a hypoxia-inducible factor 1 inhibitor, selectively induces apoptosis in hypoxic cells through depolarization of mitochondria in A549 human lung cancer cells. Mol Med Rep.

[25] Liang CC, Park AY, Guan JL (2007). *In vitro* scratch assay: a convenient and inexpensive method for analysis of cell migration *in vitro*. Nat Protoc.

[26] Tamaki N, Hirata K (2016). Tumor hypoxia: a new PET imaging biomarker in clinical oncology. Int J Clin Oncol.

[27] Fleming IN (2015). Imaging tumour hypoxia with positron emission tomography. Br J Cancer.

[28] Winnard PT (2008). Molecular imaging of metastatic potential. J Nucl Med.

[29] Wang H (2009). Using dual-tracer PET to predict the biologic behavior of human colorectal cancer. J Nucl Med.

[30] Deng YJ, Qiu HM (1998). Comparison of metastatic potentials of human colorectal carcinoma cell lines and their features related to metastasis. World Chinese Journal of Digestology.

[31] Lu X, Kang Y (2010). Hypoxia and hypoxia-inducible factors: master regulators of metastasis. Clin Cancer Res.

[32] Kudo T (2011). PET imaging of hypoxia-inducible factor-1-active tumor cells with pretargeted oxygen-dependent degradable streptavidin and a novel 18F-labeled biotin derivative. Mol Imaging Biol.

[33] Sato J (2013). 18F-fluoromisonidazole PET uptake is correlated with hypoxia-inducible factor-1alpha expression in oral squamous cell carcinoma. J Nucl Med.

[34] Kawai N (2014). Correlation between (1)(8)F-fluoromisonidazole PET and expression of HIF-1alpha and VEGF in newly diagnosed and recurrent malignant gliomas. Eur J Nucl Med Mol Imaging.

