# Supplementary material for: Noninvasive evaluation of 18F-FDG/18F-FMISO-based Micro PET in monitoring hepatic metastasis of colorectal cancer
Source: Sci Rep. 2018 Dec 13;8:17832. doi: 10.1038/s41598-018-36238-x (PMC6292879; doi:10.1038/s41598-018-36238-x)

Supplementary Information

Title: Noninvasive evaluation of 18F-FDG/18F-FMISO-based Micro PET in monitoring hepatic metastasis of colorectal cancer

Authors: Mingyu Zhang, Huijie Jiang, Rongjun Zhang, Hailong Xu, Hao Jiang, Wenbin Pan, Xin Li, Yiqiao Wang, Song Wang

Tracking number: **SREP-18-18522**

GAPDH western blot

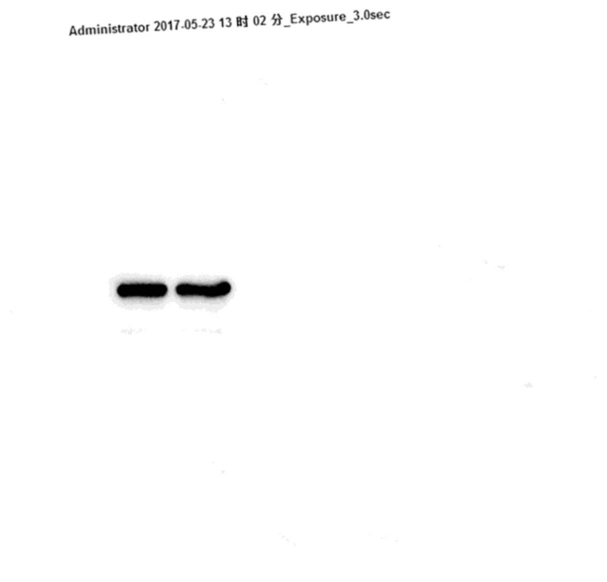

GLUT-1 western blot

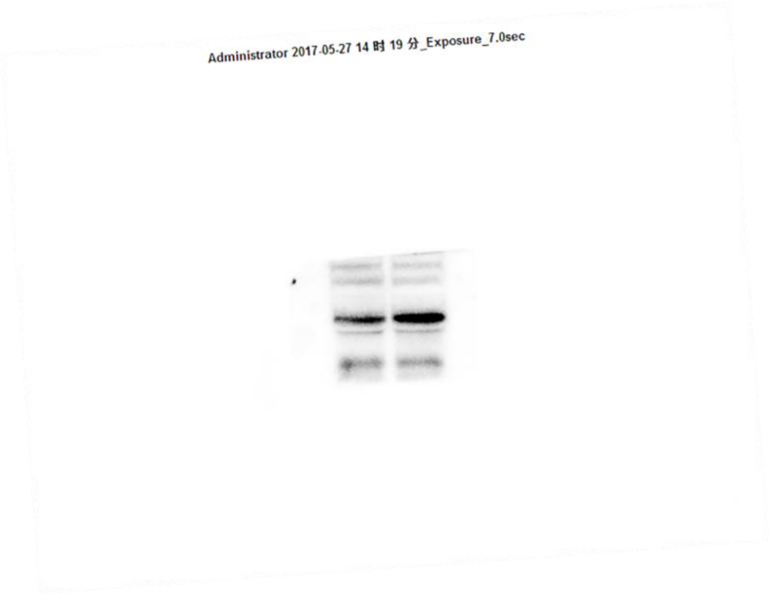

HIF-1a western blot

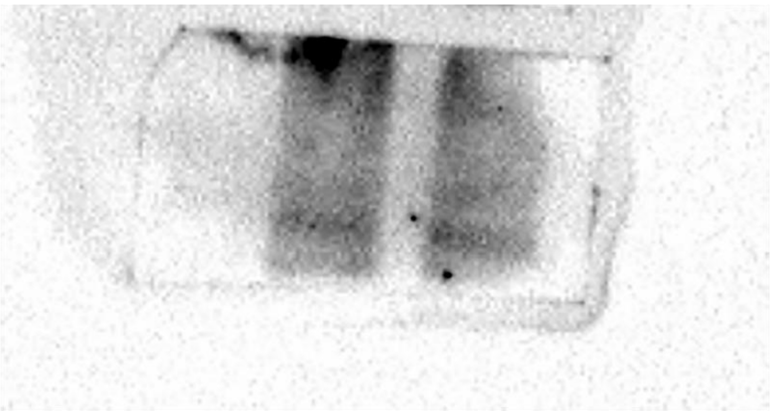

Supplement: Supplementary file 1 — Supplementary Information [file 41598_2018_36238_MOESM1_ESM.pdf]
